# *In vitro* antibacterial activity of selected medicinal plants traditionally used in Vietnam against human pathogenic bacteria

**DOI:** 10.1186/s12906-016-1007-2

**Published:** 2016-01-27

**Authors:** Thuy Thu Vu, Hyungrok Kim, Vu Khac Tran, Quang Le Dang, Hoa Thi Nguyen, Hun Kim, In Seon Kim, Gyung Ja Choi, Jin-Cheol Kim

**Affiliations:** 1 Department of Green Chemistry and Environmental Biotechnology, Korea University of Science and Technology, 217 Gajeong-Ro, Yuseong-Gu, Daejeon, 305-333 Republic of Korea; 2 Division of Bio and Drug Discovery, Korea Research Institute of Chemical Technology, 141 Gajeong-Ro, Yuseong-Gu, Daejeon, 305-600 Republic of Korea; 3 Division of Green Chemistry and Engineering, Korea Research Institute of Chemical Technology, 141 Gajeong-Ro, Yuseong-Gu, Daejeon, 305-600 Republic of Korea; 4 Department of Pharmaceutical Chemistry and Pesticides Technology, School of Chemical Engineering, Hanoi University of Science and Technology, No. 1 Dai Co Viet, Hai Ba Trung, Hanoi, Vietnam; 5 Research and Development Center of Bioactive Compounds, Vietnam Institute of Industrial Chemistry (VIIC), No. 2 Pham Ngu Lao, Hoan Kiem, Hanoi, Vietnam; 6 Division of Applied Bioscience and Biotechnology, Institute of Environmentally Friendly Agriculture, College of Agriculture and Life Sciences, Chonnam National University, 77 Yongbong-Ro, Buk-Gu, Gwangju, 500-757 Republic of Korea

**Keywords:** Antibacterial activity, Infectious diseases, Bacterial infections, Medicinal plants, Vietnam

## Abstract

**Background:**

Medicinal plants are widely used for the treatment of different infectious diseases. Infectious diseases caused by bacteria have a large impact on public health. This study aimed to determine the in vitro antibacterial activity of the medicinal plants traditionally used in Vietnam against the bacterial strains associated with infectious diseases.

**Methods:**

Methanol extracts of twelve Vietnamese medicinal plants were tested for their antibacterial activity against five bacterial species including Gram-positive bacteria (*Bacillus cereus*, *Bacillus subtilis*, and *Staphylococcus aureus*) and Gram-negative bacteria (*Escherichia coli* and *Pseudomonas aeruginosa*) using the broth microdilution method.

**Results:**

All the plant extracts showed antibacterial activity, especially against Gram-positive bacteria (*Bacillus cereus, Bacillus subtilis,* and *Staphylococcus aureus*). *Baeckea frutescens* extract revealed a potent activity against the Gram-positive bacteria with the minimum inhibitory concentration (MIC) and minimum bactericidal concentration (MBC) of 62.5 μg/ml. High activity against all the three Gram-positive bacteria was also observed for the extracts of *Cratoxylum formosum* ssp. *pruniflorum, Pogostemon cablin,* and *Pedilanthus tithymaloides* with MICs of 125, 125 and 250 μg/ml and MBCs of 125–250, 125–250 and 250–500 μg/ml, respectively. The extracts of *C. formosum* ssp. *pruniflorum* and *P. tithymaloides* showed a broad-spectrum antibacterial activity against all the bacteria tested with the MICs of 125–2,000 μg/ml.

**Conclusion:**

This study indicates clear evidence supporting the traditional use of the plants in treating infectious diseases related to bacteria. In particular, these plant species showed moderate to high antibacterial activity against the Gram-positive bacteria tested.

## Background

Since the mid-1970s, the emergence of a number of new pathogens and reemergence of older diseases has highlighted the fact that, contrary to expectations, epidemics of infectious disease remain a problem of public health concern [1]. Infectious diseases remain the largest global cause of death [1, 2]. They account for approximately one-half of all the deaths in tropical countries [2]. Many of these diseases are difficult to treat or have no specific effective therapy available. For most of these diseases, no vaccines are available [3]. Infectious diseases caused by bacteria have a large impact on public health [3, 4]. In recent years, the emergence of antibiotic resistance and the failure of chemotherapy are increasing [5]. Therefore, nowadays, the discovery of new natural antibacterial agents for treating infectious diseases is essential to prevent the spread of diseases and improve their treatment.

Similar to microorganisms, plants are a biologically and chemically diverse resource. It is estimated that there are 250,000 to 500,000 species of plants on the Earth [6]. Plants have been used as traditional medicines for the treatment of various diseases throughout most of human history. The use of plant extracts as medicinal treatments gained popularity in the late 1990s [7]. Plants are still an important source of medicines, especially in developing countries where the plant-based traditional medicines are still used to meet the health-care needs [8].

Despite the recent interest in drug discovery using molecular modeling, combinatorial chemistry, and other synthetic chemistry methods, natural product-derived compounds are still proving to be an invaluable source of medicines for humans [8]. Several recent studies have shown the increased interest in plant materials for their diverse pharmacological and biological properties including antibacterial activity [2, 9–12].

This study was aimed at validating the traditional use of selected Vietnamese medicinal plants against common bacteria, causing several human infections including *Escherichia coli*, *Pseudomonas aeruginosa*, *Bacillus cereus*, *Bacillus subtilis*, and *Staphylococcus aureus* [2, 4, 13], by evaluating their in vitro antibacterial activity. The plants investigated in this study commonly used to treat the infectious diseases and the associated symptoms are listed in Table 1.

**Table 1:** Selected Vietnamese medicinal plants used for treatment of infectious diseases

Plant species (family); Voucher number/place of collection	Common name	Parts used	Traditional uses [20]	Previous reports on antibacterial activities
*Aglaia odorata* Lour. (Meliaceae); THTV2/Ha Tay	Ngâu dại	Leaves, flowers	Fever, jaundice, asthma, scabies	Stem oil and ethanolic extract: Bc, Sa [29].
*Baeckea frutescens* L. (Myrtaceae); THT136/Thua Thien Hue	Chổi xuể	Leaves, flowers	Influenza, indigestion, sores, menstrual irregularities	Leaf essential oil and its emulsion: Sa [21]. Plant methanolic extract: Sm [22].
*Cannabis sativa* L. (Cannabaceae); THT26/Lam Dong	Gai dầu	Top with flowers and fruits	Pain killers, antiseptic, burn treatments	Cannabinoids compounds isolated from plant extract: MRSA [30]. Essential oil of aerial part [31]: Sa, Sm, Bs, Stm. Plant methanolic and hexane extracts: Bs, Bc, Ec, Pa, St [32].
*Cassytha filiformis* L. (Lauraceae); TH107/Thua Thien Hue	Tơ hồng	Trunk	Cough, fever, tonic medicine, blood purification, gonorrhea	Methanolic extract of aerial part: Ec, Pa [33].
*Cinnamomum camphora* (L.) J. Presl (Lauraceae); 445/Ha Tay	Long não	Wood, leaves, roots	Abdominal pain, anticeptic, antiinflammatory	Camphor-a major component of essential oil: biological properties [34].
*Citrus grandis* (L.) Osbeck (Rutaceae); 608/Vinh Phuc	Bưởi	Leaves, fruit peel	Influenza, headache, abdominal pain, cough, indigestion	Fruit tissue extracts and isolated compounds: Bc, Bs, Sa, Ec, Se [35, 36]. Carotenoids extract of flavedo: Bs, Sa, Ec [37].
*Clematis vitalba* L. (Ranunculaceae); BTC10/Gia Lai	Mộc thông	Stem and roots	Indigestion, diuretic, galactagogue	/
*Cratoxylum formosum* subsp. *pruniflorum* (Kurz) Gogelein (Clusiaceae); THT190/Cao Bang	Thành ngạnh	Leaves	Aid digestion, ailment	Isolated compounds from roots and barks: Bs, Sa, Pa, Sf, St, Ss [23].
*Euphorbia hirta* L. (Euphorbiaceae); THT19/Ha Noi	Cỏ sữa lá lớn	Whole plant	Dysentery	Organic extracts of aerial parts: Ec, Pa, Sa, Ss, St, Ea, Kp, Pm, Pv, Sd [38]. Flavonoids extracts of different parts: Ec, Pa, Sa, Pm [39].
*Pedilanthus tithymaloides* (L.) Poit (Euphorbiaceae); BTC40/Ha Noi	Thuốc giấu	Leaves	Wounds	Ethanolic, methanolic extracts and isolated compound of leaf: Sa, Bs, Ec, Pa, Sd [24, 25].
*Pluchea indica* (L.) Less. (Compositae); THTV3/Ha Noi	Cúc tần	Leaves, shoots, and roots	Fever, influenza, dysentery, aid digestion	Aqueous extract of aerial part: Ec, Kp [40]. Isolated compound from root: Sa, Bs [41].
*Pogostemon cablin* (Blanco) Benth. (Lamiaceae); THT20/Ha Tay	Hoắc hương	Leaves and twigs	Influenza, headache, aid digestion	Hexane extract of leaf: Ec, Bs, Sa, Ea [26]. Essential oil and isolated compounds: Ec, Pa, Sd, Sa, Tb, Bs, Ste [27, 28].

Bc: *Bacillus cereus*; Bs: *Bacillus subtilis;* Ea: *Enterobacter aerogens*; Ec: *Escherichia coli*; Kp: *Klebsiella pneumonia*; MRSA: Methicillin-resistant *Staphylococcus aureus*; Pa: *Pseudomonas aeruginosa*; Pm: *Proteus mirabilis*; Pv: *Proteus vulgaris*; Sa: *Staphylococcus aureus*; Sd: *Shigella dysenteriae*; Se: *Salmonella enteritidis*; Sf: *Streptococcus faecalis*; Sm*: Streptococcus mutans*; Sp: *Bacillus proteus*; Ss: *Shigella sonei*; St: *Salmonella typhi*; Ste: *Staphylococcus epidermidis*; Stm: *Salmonella typhimurium*; Tb: *Typhoid bacillus*; (/): not reported

## Methods

### Plant materials and extraction

The plants were collected from different locations in Vietnam between March and October 2012. The collected species were authenticated by Associate Prof. Vu, Xuan Phuong, Dr. Tran, The Bach, and Dr. Nguyen, The Cuong from Institute of Ecology and Biological Resources, Vietnam. Voucher specimens of the plants were deposited in the Herbarium of the Department of Phytochemistry and Research and Development Center of Bioactive Compounds, Vietnam Institute of Industrial Chemistry (VIIC). The collected plant materials were air-dried and finely powdered using a blender. To prepare methanol extracts of the plant materials, 10 g of each powdered plant material was extracted twice with 100 ml of methanol for 48 h at room temperature. The extracted suspensions were filtered through Whatman No. 1 filter paper, and the filtrates were concentrated to dryness using a rotary evaporator, and then stored at −20 °C until further use. For the antibacterial activity assays, the extracts were dissolved in dimethyl sulfoxide (DMSO) at a concentration of 100 mg/ml and stored at 4 °C as stock solutions.

### Bacterial strains and culture conditions

Five bacterial species including two Gram-negative (*Escherichia coli* American Type Culture Collection, ATCC 25922 and *Pseudomonas aeruginosa* ATCC 9027) and three Gram-positive strains (*Bacillus cereus* ATCC 21768, *Bacillus subtilis* ATCC 6633, and *Staphylococcus aureus* ATCC 6538) were obtained from the ATCC (Manassas, VA, USA). The bacterial strains were cultured aerobically on nutrient agar (NA) plates at 37 °C for 24 h. For the antibacterial activity test, the bacteria were aerobically cultured in nutrient broth (NB) at 37 °C for 24 h, and then suspended in sterile saline at a density equivalent to that of the 0.5 McFarland standard. Bacterial suspensions with a concentration of 10^5^ cfu/ml were used for in vitro antibacterial activity test.

### Determination of antibacterial activity

The minimum inhibitory concentration (MIC) of each plant extract was determined using the broth microdilution method as described in our previous report [14]. Briefly, two-fold serial dilutions of each plant extract were added to the wells of sterile 96-well plates containing inoculated NB medium (100 μl) with bacterial cells (10^5^ cfu/ml). The final concentrations of the plant extracts ranged from 15.63 to 2,000 μg/ml. DMSO (2 %) was used as the negative control, which did not affect the bacterial growth. Streptomycin sulfate and chloramphenicol (Sigma-Aldrich, USA) were used as positive controls against all the bacteria. Following a 24–h incubation at 37 °C, the MIC was determined as the lowest concentration that completely inhibited the growth of the bacteria. The assay was repeated twice with two replicates for each extract against the individual bacterial species at all the test concentrations.

For the poorly water-soluble extracts, the MIC was determined according to the previously described method of Kurekci et al. with some modification [15]. Iodonitrotetrazolium chloride (20 μl, 0.2 mg/ml, INT, Sigma-Aldrich) was added to the test wells at the completion of the incubation period, and this was further incubated at 37 °C for 3 h. The presence of viable bacteria was determined based on the dye changing color from yellow to pink.

The minimum bactericidal concentration (MBC) of each extract was determined by withdrawing 20 μl of the bacterial broth suspension showing no color change, and then spreading it on NA plates, which were then incubated at 37 °C for 24–48 h. The lowest concentration of the extract at which no bacterial growth was observed, was considered as the MBC.

## Results and discussion

In this study, 12 plants from 10 different families were selected according to their traditional usage for the treatment of infectious diseases and associated symptoms. The identification of the studied plants by their scientific and common names, traditional use, parts used, place of collection and voucher number is listed in Table 1. All the plants were extracted with methanol, because methanol is considered as the best solvent for the extraction of antimicrobial substances and may contain diverse chemical compounds with biological activity [2, 16].

The in vitro antibacterial activity of plants was evaluated against both Gram-positive and negative strains using the microdilution method to determine their MIC and MBC values. The MIC and MBC values of the extracts from various plant species against test bacteria are listed in Table 2. Most of the extracts exhibited a broad antibacterial spectrum against the strains tested. The extracts of *Cratoxylum formosum* ssp. *pruniflorum* and *Pedilanthus tithymaloides* displayed broad-spectrum antibacterial activities against all the five strains tested with the MIC values in the ranges 125–2,000 and 250–2,000 μg/ml, respectively. The extracts of *Pogostemon cablin* and *Pluchea indica* showed the MIC values in the ranges 125–2,000 and 500–2,000 μg/ml, respectively, against four bacterial strains except *P. aeruginosa*. On the other hand, streptomycin sulfate and chloramphenicol used as positive controls showed strong antibacterial activities against both Gram-positive and Gram-negative bacteria like as the results of previous studies [17–19].

**Table 2:** Antibacterial activity of medicinal plant extracts against common bacterial strains

Plant species	Parts used	MIC (MBC) (μg/mL)				
		Gram-negative bacteria		Gram-positive bacteria		
		*E. coli*	*P. aeruginosa*	*B. cereus*	*B. subtilis*	*S. aureus*
*Aglaia odorata*	Leaves	-	-	500 (500)	1,000 (2,000)	500 (1,000)
*Baeckea frutescens*	Leaves	-	-	62.5 (62.5)	62.5 (62.5)	62.5 (62.5)
*Cannabis sativa*	Leaves and branches	-	-	2,000 (>2,000)	-	2,000 (>2,000)
*Cassytha filiformis*	Trunk	-	-	2,000 (>2,000)	-	2,000 (>2,000)
*Cinnamomum camphora*	Leaves and branches	-	-	250 (250)	125 (125)	500 (1,000)
*Citrus grandis*	Leaves	-	-	500 (1,000)	1,000 (2,000)	1,000 (2,000)
*Clematis vitalba*	Stem	-	-	250 (250)	500 (500)	500 (1000)
*Cratoxylum formosum* ssp. *pruniflorum*	Leaves	1,000 (2,000)	2,000 (>2,000)	125 (125)	125 (250)	125 (250)
*Euphorbia hirta*	Whole plant	-	-	500 (1,000)	250 (250)	500 (1,000)
*Pedilanthus tithymaloides*	Leaves	2,000 (>2,000)	2,000 (>2,000)	250 (250)	250 (500)	250 (250)
*Pluchea indica*	Leaves	2,000 (>2,000)	-	500 (500)	1,000 (1,000)	500 (500)
*Pogostemon cablin*	Leaves and branches	2,000 (>2,000)	-	125 (250)	125 (125)	125 (250)
Streptomycin sulfate		10 (10)	10 (20)	5 (10)	2.5 (5)	5 (10)
Chloramphenicol		1.25 (2.5)	80 (80)	2.5 (2.5)	0.63 (1.25)	5 (5)

*E. coli*: *Escherichia coli*; *P. aeruginosa*: *Pseudomonas aeruginosa*; *B. cereus*: *Bacillus cereus*; *B. subtilis: Bacillus subtilis; S. aureus: Staphylococcus aureus*; (-): MIC not detected at up to 2,000 μg/mL

Most of the plant extracts tested displayed impressive antibacterial efficacies against Gram-positive bacteria with the MIC values ≤ 1,000 μg/ml. In particular, the methanol extract of the leaves and branches of *Baeckea frutescens* showed a potent antibacterial activity against all the Gram-positive bacteria tested with the MIC and MBC values of 62.5 μg/ml each. High activity was also observed for the extracts of *C. formosum* ssp. *pruniflorum* and *P. cablin* (MIC, 125 μg/ml each and MBC, 125 and 250 μg/ml, respectively) followed by the extracts of *Cinnamomum camphora* and *P. tithymaloides* (MIC, 125–500 μg/ml and MBC values of 125–1,000 μg/ml). The extracts of *Cannabis sativa* and *Cassytha filiformis* were less active against the bacterial strains tested. In contrast, the antibacterial activity against the Gram-negative bacteria was shown only by some extracts with the MIC values of 1,000 and 2,000 μg/ml (Table 2). The results obtained in this study indicate that the Gram-negative bacteria were less susceptible to the plant extracts than the Gram-positive bacteria.

The antibacterial activities of most of the plants evaluated in this study were previously tested against a few bacterial strains as listed in Table 1. However, not all of them were tested against the strains used in this study. Interestingly, no previous study has reported the antibacterial activity of *Clematis vitalba*, which showed a good activity against the Gram-positive bacteria with the MIC values in the range 250–500 μg/ml. To the best of our knowledge, this study is the first report on the antibacterial activity of *C. vitalba*.

The methanol extract of *B. frutescens* exhibited the most potent antibacterial activity with the MIC values of 62.5 μg/ml against the Gram-positive bacteria (Table 2). Traditionally, decoction of leaves and flowers of this plant has been used to treat menstrual irregularities and essential oil of the plant to treat symptoms such as influenza, sores, and indigestion [20]. The plant was also used as a medicinal plant in China, Malaysia, and Indonesia [21]. Its antibacterial activity was reported in some previous studies against only *Streptococcus mutans* and *S. aureus* [21, 22]. However, detailed information on the antibacterial properties of *B. frutescens* is lacking.

*C. formosum* ssp. *pruniflorum* belongs to the family Clusiaceae. The *Cratoxylum* genus distributed in several Southeast Asian countries. Some species of this genus have been used for the treatment of diuretic, stomachic and tonic effects, as well as diarrhea and food poisoning [23]. This plant has been used traditionally to aid digestion and treat ailments in Vietnam [20]. Plants of this genus produce various types of secondary metabolites, including xanthones, triterpenoids, and flavonoids. Some compounds such as prenylated xanthones and anthraquinones isolated from the roots and barks of this plant showed strong antibacterial activities against several bacteria including *B. subtilis, S. aureus*, and *P. aeruginosa* [23]. In this study, the methanol extract of the leaves of *C. formosum* ssp. *pruniflorum* also displayed a broad-spectrum and potent antibacterial activity against all the bacterial strains tested. The antibacterial activity of this extract is likely to be associated with the presence of these compounds.

This study also indicates that methanol extract of *P. tithymaloides* leaves possesses a broad-spectrum antibacterial activity against all the test bacterial strains. The previous studies of Vidotti et al. [24] and Ghosh et al. [25] showed the same behavior of its methanol and ethanol extracts against *B. subtilis, S. aureus, E. coli*, and *P. aeruginosa,* but the methanol extract in our study is less effective against the Gram-negative strains.

In this study, the methanol extract of *P. cablin* was found to be active against all the bacterial strains tested except *P. aeruginosa*. However, polar solvent extracts (ethanol, methanol, and aqueous) of this plant did not form growth inhibition zones against *S. aureus, E. coli*, and *P. aeruginosa* by the disc diffusion method, whereas its hexane extract did [26]. In addition, essential oil and its major compounds from this plant showed a broad-spectrum antibacterial against various bacterial strains including *B. subtilis, S. aureus, E. coli,* and *P. aeruginosa* [27, 28]. The results of this study are in agreement with the literature reports.

Most of the previous studies reported the use of different methods or bacterial species, therefore, a direct comparison of literature data with our present study was difficult. The contrasting results might be largely attributed to the different locations where the plants were collected and the solvent used in the extraction.

## Conclusions

All the plant species evaluated in this study are currently used traditionally for the treatment of various infectious diseases (Table 1), and showed promising antibacterial activity against the Gram-positive bacteria including *B. cereus, B. subtilis,* and *S. aureus*. The antibacterial activity of *B. frutescens* was highly significant with the MIC values of 62.5 μg/ml. Furthermore, this is the first study to report the antibacterial activities of *C. vitalba*. Further phytochemical studies are necessary to provide relevant information for the development of these plants as potential effective treatments against bacterial infections and diseases. Finally, the results of this study clearly elucidate the antibacterial activities of these plants and provide an evidence to support their use in folk medicine.
